# How within-country inequalities and co-coverage may affect LiST estimates of lives saved by scaling up interventions

**DOI:** 10.1186/1471-2458-13-S3-S24

**Published:** 2013-09-17

**Authors:** Cesar G Victora, Aluisio J D Barros, Tanya Malpica-Llanos, Neff Walker

**Affiliations:** 1 Post-Graduate Program in Epidemiology, Universidade Federal de Pelotas, Brazil; 2 Department of International Health, Johns Hopkins University Bloomberg School of Public Health, Baltimore, MD, USA

## Abstract

Lives-saved estimates calculated by LiST include the implicit assumptions that there are no inequalities among different socioeconomic groups, and also that the likelihood of a mother or child receiving a given intervention is independent from the probability of receiving any other interventions. It is reasonable to assume that, as a consequence of these assumptions, LiST estimates may exaggerate the numbers of lives saved in a population, by ignoring the fact that coverage is likely to be lower and mortality higher among the poor than the rich, and also by failing to take into account that coverage with different interventions may be clustered at individual mothers and children – a phenomenon described as co-coverage. We used data from 127 DHS surveys to estimate how much these two assumptions may bias estimates produced by LiST, and conclude that under real-life conditions bias occurred in both directions, with LiST results either over or underestimating the more complex estimates. With few exceptions, bias tended to be small (less than 10% in either direction).

## Introduction

The Lives Saved Tool, or LiST (http://www.jhsph.edu/departments/international-health/IIP/list/) is being increasingly used to estimate the impact of changes in intervention coverage on the mortality of children under five years of age [1-3]. LiST is a computer-based tool that estimates the impact of scaling up interventions on maternal, neonatal and child mortality. The main purpose of this tool is to promote evidence-based program planning for maternal, neonatal, and child health [4]. The model works by establishing a baseline description of the country or region in terms of demographic information, nutritional status, causes of deaths and levels of risk factors and exposure variables (levels of intrauterine growth restriction, breastfeeding patterns and rates by age, percent of the population exposed to falciparum), and current coverage of over 60 interventions. Each intervention is also defined in terms of its efficacy in reducing one or more causes of death. The model allows users to create various scenarios where they can scale up different intervention packages and estimate the impact of the various scenarios of maternal, neonatal and child mortality.

There have been numerous comparisons to the modeled impact from LiST to measured mortality reduction (e.g., [1-3,5]). Overall, these comparisons suggest that in general LiST can be used to estimate the impact of scaling up interventions on neonatal and child mortality. In this article we explore how two separate issues may affect the validity of national LiST estimates.

First, the algorithm used in LiST to estimate mortality ignores the fact that children who are most likely to die (e.g. those from the poorest families) may also be less likely to receive life-saving interventions [6]. If intervention coverage increases more rapidly among the rich, who were less likely to die at baseline, the impact estimated by LiST may be biased upwards. We will investigate this by using household asset indices to stratify national survey samples into wealth quintiles, and then run LiST separately in each quintile for selected countries. We will calculate the number of deaths prevented by scaling up selected interventions using two approaches: (a) assuming that they are equally scaled up in all quintiles (as LiST currently does) and (b) carrying out separate simulations for each quintile using actual increases in coverage, and subsequently aggregating the number of deaths prevented in the whole population. The comparison of the two sets of results will allow us to estimate how much bias may arise from ignoring within-country inequalities.

The second issue is that LiST assumes independent probabilities of being covered by different interventions; for example, a mother who receives antenatal care is just as likely to have her child delivered by a skilled attendant as a mother who did not receive antenatal care. This is unlikely to be true in the real world, where interventions will tend to be clustered at the mother/child level, a finding that has been described as “co-coverage” [7]. Lack of independence among several interventions is likely to lead to an overestimate of the number of lives saved, given that the increase in coverage may be concentrated on children already receiving other interventions and under a lower mortality risk. We will perform simulations using existing datasets to assess the magnitude of co-coverage and how this may affect LiST estimates.

We tested these two potential sources of bias using data from the Countdown to 2015 Equity Database [8,9]. To our knowledge this is the first examination of these issues in the literature.

## Methods

Different data and approaches were used in the two sets of analyses.

### Effect of wealth inequalities

We listed all Demographic Health Surveys (DHS) and Multiple Indicator Cluster Surveys (MICS) carried out in Countdown countries since 1990 that included data on household assets. Thirty five countries had two or more surveys with an interval of 5-13 years between them. If a country had only two such surveys, we selected these two. If it had three or more, we gave priority to selecting two surveys of the same type (i.e. two DHS or two MICS) in order to increase comparability of results. If there were three surveys of the same type, we chose the earliest and the most recent, in order to maximize the number of years between them. Of the 35 countries, 23 were in sub-Saharan Africa, eight in Asia, three in Latin America and the Caribbean and one in North Africa. This database has been recently used in analyses of changes in inequalities over time [10].

We selected two interventions, each of which is effective against a single cause of death. We avoided interventions that are effective against several causes of death, such as antenatal care, skilled birth attendant or breastfeeding, because these are more difficult to model and involve a larger number of assumptions. The first intervention, care-seeking for pneumonia, has been present for a long time and shows important inequalities in most countries [11]. The second represents a recently introduced intervention, insecticide treated net use by children (ITNs) in malaria-endemic countries, for which coverage is increasing rapidly in many countries.

A household-level wealth index was used to stratify mothers and children according to socioeconomic position, as calculated by the original DHS or MICS survey [12]. The wealth index was divided into quintiles, with Q1 representing the poorest 20% of households and Q5 representing the richest. Individuals were classified according to the quintile of the household where they lived.

To ensure full compatibility between the quintile-specific and national estimates of mortality and coverage, we used for the latter the weighted average of the quintile-specific levels, instead of the aggregate values obtained from the national analyses. The differences between the two sets of national values were very small, less than 2 percent points for the large majority of comparisons.

We did six separate LiST runs, one for each quintile and one for the whole population. We then summed the number of lives saved in the five quintiles to obtain the adjusted estimate. Then, this adjusted estimate was compared to the aggregate estimate (ignoring the quintiles) using a bias indicator calculated as follows:

This approach assumes that the equity-stratified estimates are the true values, and differences between these and the traditional LiST estimates constitute bias. If bias was greater than one, traditional analyses are over-estimates. If smaller than one, underestimates. In the table below, we express bias as a percentage, after subtracting 1 from the above formula.

To examine the magnitude of bias resulting from ignoring wealth quintile differences when performing LiST estimates, we used the following parameters for the 35 countries:

1. We obtained estimates of intervention coverage by quintile, for the baseline and endline surveys.

2. We obtained estimates of under-five mortality by quintile from the baseline survey.

3. We obtained the number of births by quintile, also from the baseline survey (quintiles were calculated for households, and due to higher fertility rates, usually there are more mothers and children in the poorer quintiles than in the richer quintiles).

4. We used country-specific proportions of deaths due to pneumonia and malaria, as calculated by the Child Health Epidemiology Reference Group [13].

5. We used the efficacy data included in the LiST software to estimate lives saved by pneumonia care-seeking [14] and ITNs [15].

6. Slope indices of inequality were calculated for coverage change over time. These express the absolute difference, in percent points (p.p.), between subjects at the top and bottom of the wealth scale [16]. Positive values show that coverage increases were larger among the rich than for the poor.

As a consistency check, we followed exactly the same procedures described above using an Excel spreadsheet, and compared the results with those obtained with LiST.

### Effect of co-coverage

We analyzed data from 127 DHS surveys from 60 countries, that were available by early 2012. A full list of surveys and countries is available in Additional file 1. Within each survey, the units of analyses were mother-child pairs, and we calculated correlation coefficients for all possible combinations of six coverage indicators, all coded as 0 or 1:

- ANC: antenatal care (4 or more visits),

- SBA: skilled birth attendant,

- DPT: three doses of diphtheria, pertussis and tetanus vaccine,

- WAT: improved water source,

- VTA: vitamin A supplementation in the previous six months, and

- ITN: insecticide treated net use by the child.

We used the results from the correlation analyses to run a series of simulations using Excel. The simulations were carried out with several assumptions. We set mortality due to causes preventable by ANC or SBA at 50 per thousand births. For simplicity we set baseline coverage levels with both interventions to 0%, and increased it to 100%. We set efficacy of ANC at 0.2 (20% reduction in mortality with universal coverage) and SBA efficacy at 0.3, and their joint efficacy at 0.44, that is, 20% of deaths would be prevented by ANC and of the remaining 80% of deaths, 30% (or 24% of the total number of deaths) would be prevented by SBA, if both interventions were scaled up to 100% coverage. This modeling exercise assumes that there is neither synergism nor antagonism between SBA and ANC. It should be noted that baseline coverage, mortality levels and efficacy parameters do not affect the calculation of bias because these are applied across the board, both in the traditional LiST approach and in the estimates that allow for co-coverage.

In the traditional LiST approach, we assumed independence between SBA and ANC coverage, that is, mothers receiving ANC were as likely to receive SBA as those mothers who did not receive ANC. We then estimated how many lives would be saved by scaling up coverage to 100% with both interventions.

The co-coverage approach assumed different degrees of co-coverage, that is, mothers using ANC were more likely to also receive SBA. This was done by varying the conditional probability of receiving SBA according to whether or not the mother had also received ANC, so as to reproduce different magnitudes of the correlation coefficient between the two variables. The population was then divided into the four possible combinations of ANC and SBA, the numbers in these subgroups were estimated on the basis of the conditional probabilities, and the above-described efficacy levels were applied to the four subgroups of the population, allowing us to estimate how many lives would be saved in each subgroup.

Consistently with the equity analyses, bias was defined as:

Again, if bias was greater than one, traditional analyses are over-estimates, if smaller than one, underestimates. In the table, we express bias as a percentage, after subtracting 1 from the above formula. These simulations were carried out using Excel because LiST does not currently allow for lack of independence between coverage with separate interventions.

## Results

### Effect of wealth inequalities

Of the 35 countries with two surveys, 34 had data on careseeking for pneumonia. Fourteen of these showed either declines in coverage over time or increases of less than three percent points, and were excluded from the analyses. Results for the remaining 20 countries are presented in Table 1. The equity profiles for all countries with data on careseeking for pneumonia and insecticide-treated bednet are presented in Additional file 1.

**Table 1:** Bias in LiST estimates resulting from ignoring within country inequalities, in 20 countries with increases over time in care-seeking for pneumonia.

Country	First survey	Second survey	Baseline coverage	Endline coverage	Coverage change	Slope index*	Bias
**Madagascar**	1997	2008	37%	42%	5	21	63%
**Ethiopia**	2000	2005	16%	19%	3	-8	14%
**Ghana**	1998	2008	26%	51%	25	10	5%
**Burkina Faso**	1998	2003	22%	36%	14	12	4%
**Nepal**	1996	2006	18%	43%	25	7	3%
**Mozambique**	1997	2003	39%	55%	17	-15	0%
**Rwanda**	2000	2005	16%	28%	12	-5	0%
**Bangladesh**	1996	2007	33%	57%	24	-5	-1%
**Cambodia**	2000	2010	37%	77%	40	-14	-1%
**Malawi**	2000	2010	27%	70%	44	-5	-1%
**Niger**	1998	2006	26%	47%	22	-16	-2%
**Uganda**	1995	2006	61%	73%	12	-31	-3%
**Lesotho**	2004	2009	59%	66%	7	-44	-6%
**Mali**	1995	2006	32%	38%	6	-20	-6%
**Kyrgyzstan**	1997	2005	48%	63%	15	-70	-7%
**Peru**	1996	2004	46%	71%	25	-18	-9%
**Egypt**	1995	2008	62%	73%	12	-21	-12%
**Benin**	1996	2006	32%	36%	4	-17	-15%
**India**	1998	2005	64%	70%	6	-18	-16%
**Bolivia**	1998	2008	43%	51%	8	-20	-22%

(*) Slope index of inequality for change in coverage by wealth quintile, expressing the difference in percentage points between the increase in coverage for those at the top of the wealth distribution compared to those at its bottom.

The average bias in the 20 countries was -0.6% and the median was -1.6%, indicating that the traditional approach was very similar to the equity-stratified approach overall. Bias above 10% was observed in six countries. The most extreme case was Madagascar, where bias was equal to 63%. This was a result of declines in coverage in the two poorest quintiles, in contrast with increases in the three richest quintiles. Negative bias, showing underestimation by LiST, was also present in some countries, with Bolivia being the most extreme example with a value of –22% that was associated with faster increase in coverage among the poor than for the rich.

Table 1 also shows the slope index of inequality. Positive values indicate that coverage increases were larger for the rich than for the poor, and negative values show the opposite trend. In general, countries with positive bias also had positive slope indices, with a Spearman correlation of 0.81 (p<0.001) between the two variables. Bias, on the other hand, was not associated with overall coverage change (Spearman r = 0.18; p=0.45).

We carried out similar analyses for ITNs. Of the 21 malaria-endemic countries with ITN data, four had increases in ITN lower than 3 p.p. between the two surveys; in three other countries, malaria accounted for fewer than 3% of all deaths. Both groups of countries provided highly imprecise estimates of bias, and were excluded from the analyses. Data for the remaining 14 countries are presented in Table 2.

**Table 2:** Bias in LiST estimates resulting from ignoring within country inequalities, in 20 countries with increases over time in care-seeking for pneumonia.

Country		First survey	Second survey	Coverage change	Slope index*	Bias
**CAR**		1994	2006	15	28	15%
**Cote Ivoire**		1994	2006	4	7	13%
**Senegal**		1997	2005	7	2	3%
**Benin**		1996	2006	21	30	1%
**Zambia**		1996	2007	28	10	1%
**Niger**		1998	2006	7	8	1%
**Ghana**		1998	2008	29	-3	0%
**Tanzania**		1999	2010	61	1	0%
**Mali**		1995	2006	27	7	-1%
**Togo**		1998	2006	39	-8	-1%
**Cameroon**		1998	2006	13	10	-2%
**Malawi**		2000	2010	40	27	-2%
**Madagascar**		1997	2008	45	4	-3%
**Uganda**		1995	2006	10	3	-3%

The average bias in the 20 countries was 1.4% and the median was 0.1%, indicating that the traditional approach was very similar to the equity-stratified approach overall. Bias above 10% was observed only in the Central African Republic and in Cote d’Ivoire; the traditional LiST approach overestimated impact on both countries. Negative bias, showing underestimation by LiST, was small when present.

Correlations between bias and overall coverage increase (r=-0.393; p = 0.17) and the slope index of coverage change (r=0.294; p=0.31) were not significant.

### Effect of co-coverage

Table 3 shows the mean values of the correlation coefficients across all 127 surveys with data on the relevant indicators, as well as their variability. Nearly all surveys had data on ANC, SBA, DPT and WAT. Information on VTA was available for 59 surveys, but only 18 malaria-endemic countries had data on ITN.

**Table 3:** Mean values of Pearson correlation coefficients for selected pairs of interventions, based on individual (mother-child) level analyses in 127 DHS surveys.

**Correlation**	**Surveys**	**Pearson correlation coefficients (r)**			
		
		Mean	Standard deviation	Minimum	Maximum
**SBA X ANC**	127	0.322	0.130	-0.016	0.612
**SBA X DPT**	124	0.191	0.114	-0.058	0.526
**SBA X WAT**	126	0.126	0.105	-0.234	0.416
**SBA X VTA**	59	0.091	0.085	-0.057	0.397
**SBA X ITN**	18	0.078	0.068	-0.024	0.218
**ANC X DPT**	125	0.191	0.109	-0.081	0.469
**ANC X WAT**	126	0.088	0.081	-0.126	0.408
**ANC X VTA**	59	0.090	0.082	-0.042	0.395
**ANC X ITN**	18	0.058	0.039	-0.022	0.126
**DPT X WAT**	124	0.071	0.078	-0.258	0.316
**DPT X VTA**	59	0.147	0.111	-0.063	0.390
**DPT X ITN**	18	0.044	0.052	-0.044	0.144
**WAT X VTA**	58	0.037	0.047	-0.074	0.175
**WAT X ITN**	18	0.045	0.046	-0.081	0.113
**VTA X ITN**	17	0.040	0.027	-0.021	0.081

Most correlation coefficients were positive, as is shown by their mean values. However, at least one country had negative correlations for all possible combinations of coverage indicators, as shown by the fact that minimum values were negative.

The strongest mean correlation was between ANC and SBA, which is hardly surprising because these are often delivered at the same location by the same health workers. However, even for this combination the mean correlation coefficient was relatively weak (r=0.322). All other mean correlation values were below 0.2, indicating weak correlations.

The next step was to assess how much bias would be introduced by ignoring co-coverage in LiST, given different strengths of the correlation between any two variables, and different coverage levels.

Table 4 shows different scenarios with variable levels of co-coverage. We use SBA and ANC as examples, but the findings apply to any combination of two coverage indicators. SBA coverage was set to 20%, 40%, 60% and 80%. We then varied the level of co-coverage to reproduce three scenarios: weak (r = 0.1), moderate (r = 0.3) and strong (r = 0.5). In the 127 surveys, the mean value for ANC coverage was 56% and the mean correlation between ANC and SBA was 0.322 (range -0.016 to 0.612). We then modeled different levels of co-coverage – as assessed by Pearson correlation coefficients – between ANC and SBA, for different coverage levels with SBA (Table 4), and calculated the amount of bias that would result as a consequence of ignoring co-coverage.

**Table 4:** Simulations of bias associated with different strengths of co-coverage, using SBA and ANC as an example.

	SBA coverage				
	**20%**	**40%**	**60%**	**80%**	**Pearson r**
*Scenario 1: No co-coverage*					
**Lives saved**	7700	10400	13100	15800	0.00
*Scenario 2: Weak co-coverage*					
**Lives saved**	7840	10680	13520	16360	0.08 to 0.10
**Bias**	-1.8%	-2.6%	-3.1%	-3.4%	
*Scenario 3: Moderate co-coverage*					
**Lives saved**	7180	10360	13540	16720	0.24 to 0.30
**Bias**	7.2%	0.4%	-3.2%	-5.5%	
*Scenario 4: Strong co-coverage*					
**Lives saved**	6990	10480	13970	17460	0.40 to 0.51
**Bias**	10.2%	-0.8%	-6.2%	-9.5%	

Contrary to the original expectation, ignoring co-coverage often resulted in underestimation of the number of lives saved, rather than in overestimation. This is because - particularly when SBA coverage was high – moderate or strong co-coverage led to a high proportion of the population receiving both SBA and ANC and therefore to more lives being saved in the population as a whole than expected in the analyses where co-coverage was not modeled. That is, co-coverage per se displaced a substantial proportion of the population to the group receiving both interventions, particularly when overall SBA coverage was high.

In any case, even under these rather extreme assumptions, bias was consistently less than 10.2%.

## Discussion

To our knowledge, this is the first examination of how LiST estimates may be affected by socioeconomic inequalities in coverage and mortality, and by the fact that interventions tend to be clustered in the same children.

Our original assumptions were that there would a substantial amount of bias in LiST estimates resulting from ignoring within-population inequalities. In general, we expected LiST to overestimate the number of lives saved, because intervention coverage would increase faster among the rich, whereas most deaths occur among the poor.

We selected two interventions for analyses, each of which prevented deaths due to a single cause. We avoided complex interventions, such as ANC, SBA or breastfeeding, which provide protection against multiple causes and therefore involve more complex modeling. Care-seeking for pneumonia has been promoted for many years, shows reasonably high coverage in many countries, and tends to be quite inequitable. ITN is a low-coverage, recent intervention that is reasonably equitable [11].

Our preliminary conclusions are sobering. Bias went in both directions, and on average across all countries there was virtually none. Bias tended to be largest for pneumonia careseeking than for ITN, as expected because the former is more inequitably distributed within populations. The most extreme values in either direction were lower than 30%, either over or underestimation.

LiST includes complex calculations that take into account baseline mortality levels, baseline coverage, and change in coverage over time. Inequalities in each of these parameters could theoretically affect how much traditional LiST results might over or underestimate the true number of lives saved, obtained with equity-stratified calculations. Nevertheless, our results do not suggest that baseline coverage or coverage change over time are strongly associated with the amount of bias, which in general was small. Our recently published analyses of coverage change over time by wealth quintile in countries with two consecutive surveys [17] showed that coverage tended to increase more rapidly in the poor that in the rich over time, in most countries. Our original expectation was that coverage would increase faster among the rich, and thus lead to bias. Because the same dataset was used in the present analyses, this may explain why bias was not marked in most countries.

The second issue we addressed was that ignoring co-coverage would also bias LiST estimates. Because current versions of LiST do not allow for taking co-coverage into account, a series of simulations were performed with Excel. The assumption behind these analyses was that, because interventions will tend to be clustered at the level of individual mothers and children, the traditional LiST approach that assumes independence among interventions would overestimate the number of lives saved. Our analyses of 127 DHS surveys show that in fact most interventions show positive correlations at the mother/child level, but that correlation coefficients tend to be weak or moderate. The strongest average correlation across all combinations of six interventions was 0.32, for ANC and SBA, both of which are delivered by the same health workers in the same facilities. Our simulation exercise showed that this level of intensity for co-coverage will only result in small amounts of bias, typically of 10% or less. Contrary to the initial expectations, ignoring co-coverage could result in bias in either direction. In particular, high coverage with one intervention in the presence of moderate or strong co-coverage will displace a larger proportion of the population to the group covered by both interventions, and therefore save more lives than would be expected in the analyses that ignore co-coverage.

There are theoretical reasons for assuming that the traditional LiST approach would be biased due to ignoring within-country inequalities and co-coverage. However, the presence exercise with real data from all Countdown to 2015 countries with two consecutive surveys shows that such bias is unlikely to be important in most countries.

Taken together, the results of the present analyses suggest that neither the fact that the traditional LiST approach ignores within population inequalities, nor that it ignores co-coverage, are likely to lead to important errors in the resulting estimates in the vast majority of countries.

## Competing interests

The authors declare that they have no competing interest with this work.

## Authors’ contributions

CV and AB developed the simulations and data sets used in the analyses. TM-L and NW ran the LiST analyses. All authors were involved in writing and the final conclusions of the work.

## Supplementary Material

Additional file 1 The additional contains figures showing the observed inequalities for careseeking for pneumonia and insecticide-treated bed nets (ITN) for children. Click here for file

## Declarations

The publication costs for this supplement were funded by a grant from the Bill & Melinda Gates Foundation to the US Fund for UNICEF (grant 43386 to "Promote evidence-based decision making in designing maternal, neonatal, and child health interventions in low- and middle-income countries”). The Supplement Editor is the principal investigator and lead in the development of the Lives Saved Tool (LiST), supported by grant 43386. He declares that he has no competing interests.

This article has been published as part of *BMC Public Health* Volume 13 Supplement 3, 2013: The Lives Saved Tool in 2013: new capabilities and applications. The full contents of the supplement are available online at http://www.biomedcentral.com/bmcpublichealth/supplements/13/S3.
