# Supplementary material for: How within-country inequalities and co-coverage may affect LiST estimates of lives saved by scaling up interventions
Source: BMC Public Health. 2013 Sep 17;13(Suppl 3):S24. doi: 10.1186/1471-2458-13-S3-S24 (PMC3847580; doi:10.1186/1471-2458-13-S3-S24)
Supplement: Additional file 1 — The additional contains figures showing the observed inequalities for careseeking for pneumonia and insecticide-treated bed nets (ITN) for children. [file 1471-2458-13-S3-S24-S1.pdf]

Annex Table 1. Baseline coverage of the LiST indicators included in program scale up

| | Antenatal care | Pregnant women protected via IPT or sleeping under an ITN | Skilled birth attendance | Exclusive breastfeeding for the first six months of life | | Vitamin A Supp. | Insecticide treated bed nets or indoor residual spraying | Case mgmt of diarrhea (ORS) | Zinc for treatment of diarrhea | Case mgmt of pneumonia (oral antibiotics)[a] | Case mgmt. of malaria (ACTs) |
|---|---|---|---|---|---|---|---|---|---|---|---|
| | | | | < 1 month | 1-5 months | | | | | | |
| **All 18 Districts** | 45% | 38% | 71%[b] | 55% [b] | 28% [b] | 89% | 64% | 23% | 4% | 33% | 26% |
| **Center North** | 49% | 36% | 75% [b] | 63% [b] | 31% [b] | 88% | 62% | 22% | 1% | 25% | 21% |
| Barsalogho | 43% | 40% | 70% [b] | 62% [b] | 38% [b] | 93% [b] | 69% | 16% | 3% | 38% | 16% |
| Boulsa | 47% | 31% | 59% | 75% [b] | 23% [b] | 73% | 68% | 9% | 0% | 19% | 16% |
| Kaya | 57% | 46% | 81% [b] | 52% [b] | 31% [b] | 97% [b] | 56% | 37% | 1% | 29% | 28% |
| Kongoussi | 45% | 26% | 92% [b] | 65% [b] | 35% [b] | 94% [b] | 61% | 24% | 0% | 33% | 16% |
| **North** | 37% | 43% | 70% [b] | 49% [b] | 28% [b] | 90% [b] | 67% | 31% | 7% | 33% | 33% |
| Gourcy | 33% | 31% | 80% [b] | 53% [b] | 21% [b] | 94% [b] | 63% | 12% | 1% | 26% | 42% |
| Ouahigouya | 39% | 57% | 58% | 48% [b] | 48% [b] | 84% | 69% | 58% | 14% | 44% | 18% |
| Séguenega | 44% | 22% | 83% [b] | 59% [b] | 29% [b] | 93% [b] | 50% | 30% | 8% | 20% | 54% |
| Yako | 36% | 51% | 75% [b] | 29% [b] | 13% | 92% [b] | 69% | 14% | 3% | 32% | 32% |
| Titao | 34% | 29% | 61% [b] | 73% [b] | 38% [b] | 91% [b] | 77% [b] | 26% | 7% | 0% | 43% |
| **Acceleration w/ pneumo mgmt** | 45% | 40% | 73% [b] | 54% [b] | 29% [b] | 88% | 64% | 29% | 2% | 30% | 26% |
| **Acceleration w/o pneumo mgmt** | 38% | 36% | 74% [b] | 57% [b] | 31% [b] | 94% [b] | 66% | 14% | 4% | 32% | 30% |

[a] Case management of pneumonia was only included as a program target in Barsalogho and Gourcy

[b] Baseline coverage for the intervention exceeded target intervention coverage
